# Multiple-trait structured antedependence model to study the relationship between litter size and birth weight in pigs and rabbits

**DOI:** 10.1186/s12711-017-0288-3

**Published:** 2017-01-20

**Authors:** Ingrid David, Hervé Garreau, Elodie Balmisse, Yvon Billon, Laurianne Canario

**Affiliations:** 1 GenPhySE, INRA, INPT, ENVT, Université de Toulouse, 31326 Castanet-Tolosan, France; 2 Pectoul, INRA, 31326 Castanet-Tolosan, France; 3 GenESI, INRA, 17700 Surgères, France

## Abstract

**Background:**

Some genetic studies need to take into account correlations between traits that are repeatedly measured over time. Multiple-trait random regression models are commonly used to analyze repeated traits but suffer from several major drawbacks. In the present study, we developed a multiple-trait extension of the structured antedependence model (SAD) to overcome this issue and validated its usefulness by modeling the association between litter size (LS) and average birth weight (ABW) over parities in pigs and rabbits.

**Methods:**

The single-trait SAD model assumes that a random effect at time $t_{j}$ can be explained by the previous values of the random effect (i.e. at previous times). The proposed multiple-trait extension of the SAD model consists in adding a cross-antedependence parameter to the single-trait SAD model. This model can be easily fitted using ASReml and the OWN Fortran program that we have developed. In comparison with the random regression model, we used our multiple-trait SAD model to analyze the LS and ABW of 4345 litters from 1817 Large White sows and 8706 litters from 2286 L-1777 does over a maximum of five successive parities.

**Results:**

For both species, the multiple-trait SAD fitted the data better than the random regression model. The difference between AIC of the two models (AIC_random regression-AIC_SAD) were equal to 7 and 227 for pigs and rabbits, respectively. A similar pattern of heritability and correlation estimates was obtained for both species. Heritabilities were lower for LS (ranging from 0.09 to 0.29) than for ABW (ranging from 0.23 to 0.39). The general trend was a decrease of the genetic correlation for a given trait between more distant parities. Estimates of genetic correlations between LS and ABW were negative and ranged from −0.03 to −0.52 across parities. No correlation was observed between the permanent environmental effects, except between the permanent environmental effects of LS and ABW of the same parity, for which the estimate of the correlation was strongly negative (ranging from −0.57 to −0.67).

**Conclusions:**

We demonstrated that application of our multiple-trait SAD model is feasible for studying several traits with repeated measurements and showed that it provided a better fit to the data than the random regression model.

## Background

In genetic studies, many traits of interest are repeatedly measured over time, which gives rise to longitudinal data. The main issue when modeling such data is to account for the covariance structure of the repeated records with a limited number of parameters. A good approach to reduce the number of parameters that need to be estimated (compared with a multiple-trait model) is to use a repeatability model, which is often used because of its simplicity. However, this model assumes, too narrowly, that repeated records are expressions of the same genetic trait and that the phenotypic correlation between repeated measures is uniform. More flexible approaches have been proposed, such as random regression (RR) [1–3], character process (CP), and structured antedependence (SAD) models [4, 5]. The most commonly used approach is the RR model [6], although this approach suffers from various drawbacks, the main one being the well-known “border effect” problem [7]. CP and SAD models have been shown to fit the covariance structures better than RR models [4, 5, 8]. However, they are less often used in genetic analyses due to the lack of user-friendly software for SAD models and to the difficulty in accounting for nonstationary longitudinal data without inflation of the number of parameters or without appropriate software for CP models.

When the correlation between several longitudinal traits needs to be taken into account, modeling the covariance structure of repeated records becomes even more complicated. Once again, RR models are more often used than CP or SAD models because their extension to multivariate situations is straightforward. However, such extensions can require a large number of parameters and the drawbacks described for univariate RR models remain [9]. Multiple-trait extension of the CP model is more complex and was discussed in Jaffrézic et al. [10]. The same authors also proposed an extension of the SAD model to accommodate multiple-trait situations [9]. Nonetheless, the use of these multiple-trait CP and SAD models is still challenging because of the lack of user-friendly and readily available software. The application of the multiple-trait RR, CP and SAD approaches is relevant for a wide range of traits that are repeatedly measured over time, such as traits related to milk production (milk yield, milk composition, and somatic cell counts [11]), animal growth (body weight and feed intake) or reproduction traits (litter size and birth weight [12]).

Breeding programs in polytocous species aim at increasing the number of young weaned per female. To reach this goal, they have primarily focused on litter size [13]. However, in some selection programs, response to selection using a simple repeatability model has been low for two reasons: litter sizes at different parities were not considered as different traits [14, 15], and larger litter sizes were correlated with decreased survival of the young [16], probably because of a smaller weight and a reduced level of maturity at birth [17].

The objective of our study was to propose a new multiple-trait SAD model (freely available software) that can take correlations among several longitudinal traits into account. To illustrate the functionality of our model, we used it to analyze litter size and average birth weight in two species, pigs and rabbits.

## Methods

### Multiple-trait SAD models

Let $y_{i} \left( {t_{j} } \right)$ be the observation of animal $i$ at time $t_{j}$. All linear mixed models used to study repeated measures of $y_{i}$ over time can be decomposed as follows:

$$y_{i} \left( {t_{j} } \right) = \mu_{i} \left( {t_{j} } \right) + u_{i} \left( {t_{j} } \right) + p_{i} \left( {t_{j} } \right),$$

where $\mu_{i} (t_{j} )$ represents the fixed effects at time $t_{j}$, and $u_{i} (t_{j} )$ and $p_{i} (t_{j} )$ the genetic and pseudo-permanent animal effect random functions, with covariance functions ${\mathbf{U}}(t_{j} ,t_{{j^{{\prime }} }} )$ and ${\mathbf{P}}(t_{j} ,t_{{j^{{\prime }} }} )$, respectively. Note that this model does not include a residual term in order to help convergence and avoid identifiability problems between structured permanent and classical residual covariance matrices [18]. Thus, the residual variance was, by definition, included in the (co)variance matrix of the pseudo-permanent effects included in the model. In the single-trait situation, possible non-null covariance between random effects at different times [i.e. ${\mathbf{U}}(t_{j} ,t_{{j^{{\prime }} }} )$ and ${\mathbf{P}}(t_{j} ,t_{{j^{{\prime }} }} )$] are taken into account in the SAD approach by modeling the form of the random-effects functions. Specifically, it assumes that a random effect at time $t_{j}$ can be explained by the previous random effects (i.e. at time $t_{k} ,k < j$). For instance, for a given random effect ${\mathbf{p}}\left( t \right)$, the general form of the SAD model of order $\alpha$ is:

$${\mathbf{p}}\left( {t_{j} } \right) = \mathop \sum \limits_{s = 1}^{\alpha } \theta_{sj} {\mathbf{p}}\left( {t_{j - s} } \right) + {\mathbf{e}}\left( {t_{j} } \right),$$

where $\theta_{sj}$ is the $s$th antedependence parameter for time $t_{j}$, and ${\mathbf{e}}(t_{j} )$ is a random normally distributed effect (error term) with mean 0 and innovation variance $\sigma_{p}^{2} (t_{j} )$. To reduce the number of parameters in the SAD model, $\theta_{sj}$ and $\sigma_{p}^{2} (t_{j} )$ are assumed to be continuous functions of time: $\theta_{sj} = \sum\nolimits_{q = 0}^{{\beta_{s} }} {a_{sq} t_{j}^{q} }$ for a function of degree $\beta_{s}$ and $\sigma_{p}^{2} \left( {t_{j} } \right) = { \exp }\left( {\sum\nolimits_{q = 0}^{\gamma } {b_{q} t_{j}^{q} } } \right)$ for a function of degree $\gamma$. A single-trait SAD model is then defined by the order of the antedependence ($\alpha$), the degree of the polynomial for each antedependence parameter ($\beta_{1}$ to $\beta_{\alpha }$), and the degree of the polynomial for the innovation variance ($\gamma$) for each random effect. We will refer to single-trait SAD models as SAD $\alpha \beta_{1} \ldots \beta_{\alpha } \gamma$ [8]. For instance SAD 111 stands for a SAD model with:

$${\mathbf{p}}\left( {t_{j} } \right) = \theta_{1j} {\mathbf{p}}\left( {t_{j - 1} } \right) + {\mathbf{e}}\left( {t_{j} } \right), \; \theta_{1j} = a_{1,0} + a_{1,1} t_{j} \; {\text{and}}\; \sigma_{p}^{2} \left( {t_{j} } \right) = { \exp }\left( {b_{0} + b_{1} t_{j} } \right).$$

We propose an extension of the single-trait SAD model to the multiple-trait situation by assuming that, in addition to the within-trait antedependence relationship, a random effect of one trait can be a function of the same random effect of the other traits considered. For two traits ${\mathbf{y}}_{1}$ and ${\mathbf{y}}_{2}$, the general form of the multiple-trait SAD model of order $\alpha , \alpha^{{\prime }} , \eta , \eta^{{\prime }}$, for a given random effect ${\mathbf{p}}$, can be written as (for $j > { \hbox{max} }\left( {\alpha , \alpha^{{\prime }} , \eta , \eta^{{\prime }} } \right)$):

1 $$\begin{aligned} & {\mathbf{p}}_{1} \left( {t_{j} } \right) = \mathop \sum \limits_{s = 1}^{\alpha } \theta_{sj} {\mathbf{p}}_{1} \left( {t_{j - s} } \right) + \mathop \sum \limits_{s = c}^{\eta } \delta_{sj} {\mathbf{p}}_{2} \left( {t_{j - s} } \right) + {\mathbf{e}}_{1} \left( {t_{j} } \right), \\ & {\mathbf{p}}_{2} \left( {t_{j} } \right) = \mathop \sum \limits_{s = 1}^{\alpha '} \theta '_{sj} {\mathbf{p}}_{2} \left( {t_{j - s} } \right) + \mathop \sum \limits_{s = c'}^{\eta '} \delta '_{sj} {\mathbf{p}}_{1} \left( {t_{j - s} } \right) + {\mathbf{e}}_{2} \left( {t_{j} } \right), \\ \end{aligned}$$

where $\theta_{sj}$ and $\theta '_{sj}$ are the $s$th antedependence parameters at time $j$ for traits 1 and 2, respectively, and $\delta_{sj}$ and $\delta_{sj}^{{\prime }}$ are the $\left( {s - c + 1} \right){\text{th}}\;{\text{or}}\;\left( {s - c^{{\prime }} + 1} \right){\text{th}}$ cross-antedependence parameters at time $j$ for traits 1 and 2, respectively. Note that, in contrast to the antedependence relationship that starts at time $t_{j - 1}$, the cross antedependence relationships show greater flexibility and start at time $t_{j - c} \;(t_{{j - c^{{\prime }} }} )$, with $c \left( {c^{{\prime }} } \right)$ greater or equal to 0. Here, ${\mathbf{e}}_{1} \left( {t_{j} } \right)$ and ${\mathbf{e}}_{2} \left( {t_{j} } \right)$ are normally distributed random effects with mean 0 and innovation variance $\sigma_{p1}^{2} \left( {t_{j} } \right)$ and $\sigma_{p2}^{2} \left( {t_{j} } \right)$, respectively. Error terms ${\mathbf{e}}_{1}$ and ${\mathbf{e}}_{2}$ are assumed to be independent, except if $c > 0$ and $c^{{\prime }} > 0$ when a correlation between the two can be considered at time $t_{1}$. This constraint on the correlation between error terms ensures the identifiability of the parameters in the multiple-trait SAD model, as has been demonstrated for structural equation models (SEM). Indeed, the multiple-trait SAD model can be considered to be a specific kind of SEM [19] in which the structural parameters are functions of time. Consider, for the sake of simplicity, the simple case of no repetition per subject. A SEM with a recursive relationship between two traits ${\mathbf{y}}_{1}$ and ${\mathbf{y}}_{2}$ for animal $i$ is:

2 $$\left\{ {\begin{array}{*{20}l} {y_{1i} = {\mathbf{x}}_{1i}^{{\prime }} {\varvec{\upbeta}}_{1} + u_{1i} + \varepsilon_{1i} } \hfill \\ {y_{2i} = \lambda y_{1i} + {\mathbf{x}}_{2i}^{'} {\varvec{\upbeta}}_{2} + u_{2i} + \varepsilon_{2i} } \hfill \\ \end{array} \Leftrightarrow \left\{ {\begin{array}{*{20}l} {y_{1i} = {\mathbf{x}}_{1i}^{{\prime }} {\varvec{\upbeta}}_{1} + u_{1i} + \varepsilon_{1i} } \hfill \\ {y_{2i} = \lambda {\mathbf{x}}_{1i}^{{\prime }} {\varvec{\upbeta}}_{1} + {\mathbf{x}}_{2i}^{'} {\varvec{\upbeta}}_{2} + \lambda u_{1i} + u_{2i} + \lambda \varepsilon_{1i} + \varepsilon_{2i} } \hfill \\ \end{array} } \right.} \right.$$

Rosa et al. [20] showed that parameter identifiability in Eq. (2) is possible by assuming independency between residuals ${\varvec{\upvarepsilon}}_{1}$ and ${\varvec{\upvarepsilon}}_{2}$. Varona et al. [12] proposed an extension of the SEM that allows for unequal recursive relationships between random terms:

3 $$\left\{ {\begin{array}{*{20}l} {y_{1i} = {\mathbf{x}}_{1i}^{{\prime }} {\varvec{\upbeta}}_{1} + u_{1i} + \varepsilon_{1i} } \hfill \\ {y_{2i} = \lambda {\mathbf{x}}_{1i}^{{\prime }} {\varvec{\upbeta}}_{1} + {\mathbf{x}}_{2i}^{{\prime }} {\varvec{\upbeta}}_{2} + \lambda_{u} u_{1i} + u_{2i} + \lambda_{p} \varepsilon_{1i} + \varepsilon_{2i} } \hfill \\ \end{array} } \right.$$

Identifiability of the parameters in Eq. (3) is achieved by assuming independency between ${\mathbf{u}}_{1}$ and ${\mathbf{u}}_{2}$ and between ${\varvec{\upvarepsilon}}_{1}$ and ${\varvec{\upvarepsilon}}_{2}$ [12]. If we discard antedependency because no repetition occurs and focus on the cross-antedependency, model (3) [and (2)] is equivalent to a multiple-trait SAD model with $c > \eta$ (recursive but not simultaneous relationship), $c^{{\prime }} = 0$ and $\eta^{{\prime }} = 0$ and a polynomial cross-antedependence function of degree 0 for both the genetic and pseudo-permanent environmental effects. Terms ${\mathbf{u}}_{1}$, ${\mathbf{u}}_{2}$ and ${\varvec{\upvarepsilon}}_{1}$, ${\varvec{\upvarepsilon}}_{2}$ are equivalent to the error terms for the genetic and pseudo-permanent environmental effects of a multiple-trait SAD model. Following recommendations for identifiability in the SEM, identifiability in the SAD model is achieved by assuming independency between the error terms in Eq. (1) for both genetic and pseudo-permanent environmental effects. In the more general case of repeated measurements over time for both traits, if $c > \eta$ and $c^{{\prime }} = 1$ (recursive cross-antedependence) for random effects, the SAD model at time $t_{1}$ is equivalent to a classical multiple-trait model (where parameters are identifiable even if random effects are correlated between traits). Correlation between the error terms of random effects at time $t_{1}$ is thus permitted in the SAD model without adversely affecting parameter identifiability. Although the previous considered a simple situation with two traits, the same SAD models for each random effect, and a recursive relationship, it is straightforward to extend the reasoning to more complicated SAD models.

As for the single-trait model, (cross-)antedependence parameters and innovation variances were assumed to be continuous functions of time. The multiple-trait SAD model is then defined for two traits by the order of the antedependence for each trait $(\alpha , \alpha^{{\prime }} )$, the starting points $(c, c^{{\prime }} )$, the order of the cross-antedependence $(\eta - c + 1, \eta^{{\prime }} - c^{{\prime }} + 1)$, the degree of the polynomial for each (cross-)antedependence parameter, and the degree of the polynomial for the innovation variance of each trait $(\gamma , \gamma^{{\prime }} )$, as well as an indicator of the presence of an initial correlation between ${\mathbf{e}}_{1} \left( {t_{1} } \right)$, ${\mathbf{e}}_{2} \left( {t_{1} } \right)$. An interesting computational property of this multiple-trait SAD model is that, as is the case for the single-trait model, the inverse of the covariance matrix ${\mathbf{P}}$ can be easily calculated by the following Cholesky decomposition [21]: ${\mathbf{P}}^{ - 1} = {\mathbf{L}}^{{\prime }} {\mathbf{D}}^{ - 1} {\mathbf{L}}$, where ${\mathbf{D}}$ is a diagonal matrix with innovation variances as components, ${\mathbf{L}}$ is a lower triangular matrix with 1s on the diagonal and the negatives of the (cross-)antedependence parameters and the initial correlation ($\rho$) between ${\mathbf{e}}_{1} , {\mathbf{e}}_{2}$ as diagonal entries. For instance, for two traits, three time points, all (cross-)antedependence of order 1, $c = c^{{\prime }} = 1$ and an initial correlation $\rho$ between ${\mathbf{e}}_{1} \left( {t_{1} } \right)$ and ${\mathbf{e}}_{2} \left( {t_{1} } \right)$, the ${\mathbf{L}}$ matrix for the vector $[{\mathbf{p}}_{y1} \left( 1 \right)\; {\mathbf{p}}_{y2} \left( 1 \right)\; {\mathbf{p}}_{y1} \left( 2 \right)\; {\mathbf{p}}_{y2} \left( 2 \right)\; {\mathbf{p}}_{y1} \left( 3 \right)\; {\mathbf{p}}_{y2} \left( 3 \right)]$ is:

$${\mathbf{L}} = \left[ {\begin{array}{*{20}c} 1 & {} & {} & {} & {} & {} \\ { - \rho } & 1 & {} & {}0 & & {} \\ { - \theta_{12} } & { - \delta_{12} } & 1 & {} & {} & {} \\ { - \delta_{12}^{{\prime }} } & { - \theta_{12}^{{\prime }} } & 0 & 1 & {} & {} \\ 0 & 0 & { - \theta_{13} } & { - \delta_{13} } & 1 & {} \\ 0 & 0 & { - \delta_{13}^{{\prime }} } & { - \theta_{13}^{{\prime }} } & 0 & 1 \\ \end{array} } \right].$$

This multiple-trait SAD model can be easily fitted to data using ASReml [22], as well as the OWN Fortran program that we have developed (available online at https://zenodo.org/record/192036#.WEAYLdLhBaQ).

The orders of (cross-)antedependence and the degrees of the polynomial functions of time for (cross-)antedependence parameters and innovation variances can be selected by comparing nested models using the likelihood ratio test and by comparing non-nested models using the Bayesian or Akaike information criterion (AIC) [23, 24]. To reduce the number of models that need to be compared, we suggest selecting the specification (order and degree) of the antedependence and innovation variances by first using a single-trait SAD model and then the cross-antedependence specification by using a multiple-trait SAD model. For the single-trait SAD model step of the selection process, we suggest to start with a SAD 100, then test an increase of the degree of the function of time ($\gamma$) for the innovation variance (i.e. SAD 101), and finally test an increase of the degree of the function of time for the antedependence parameter ($\beta_{1}$) (i.e. SAD 111). This procedure is repeated until there is no additional significant improvement of the model. Then, an increase of the order of the dependence ($\alpha$) can be tested, starting with a constant second order antedependence parameter. For instance, if the last model of order 1 selected is SAD 121, the next model tested is SAD 2201. The increase of the polynome of time for the second order antedependence parameter can then be tested (i.e. SAD 2211) and so on. Using this step-by-step selection procedure, all models are nested and can be compared using the likelihood ratio test.

### Data application

The multiple-trait SAD model was applied to data from two polytocous species (pigs and rabbits) to study the relationship between the number of young born alive per litter (litter size, LS) and the average birth weight (ABW) per litter, calculated as the sum of the weight at birth of all the individuals of a litter divided by LS. The pig dataset included 4345 litters from 1817 Large White sows over a maximum of five successive farrowings. The mean LS was 12.1 ± 3.6. Piglets were weighed at birth in all litters, the mean ABW was 1514 ± 31 g. The rabbit dataset included 8706 litters from 2286 L-1777 does [25] over a maximum of five successive kindlings. Kittens were weighed at birth for 3490 litters. The mean LS and ABW were 9.5 ± 3.1 and 83 ± 14 g, respectively. The descriptive statistics of LS and ABW per parity are in Table 1.

**Table 1:** Litter size and average birth weight by parity in pigs and rabbits

Parity	Number of litters	LS (s.e.)	Number of litters	Average BW (s.e.)
*Pigs*				
1	1508	11.7 (3.4)	1508	1419 (29)
2	1134	12.0 (3.6)	1134	1565 (31)
3	861	12.6 (3.6)	861	1564 (30)
4	515	12.5 (3.5)	515	1566 (31)
5	327	12.4 (3.3)	327	1566 (31)
*Rabbits*				
1	2013	8.1 (2.7)	115	84 (17)
2	1868	9.5 (3.0)	955	85 (15)
3	1812	10.0 (3.2)	1174	83 (14)
4	1638	10.3 (3.2)	907	82 (13)
5	1375	10.0 (3.0)	339	82 (11)

Fixed effects included in the model were initially selected separately for each trait using a step-by-step descending procedure. To do this, simple models that did not take relationships between animals into account were applied to the data, nested models were then compared using the likelihood ratio test. The same within-species fixed effects were included in the genetic multiple-trait SAD model for both traits: parity (five classes), proportion of females in the litter (covariate), and the combination of year and month of delivery (121 levels) for rabbits; and parity (five classes), season of farrowing (four classes), sire breed (seven classes) and sow weight when entering the farrowing unit (covariates) for pigs. It should be noted that the contemporary group effect (constant over parities) was included in the model as a random effect for pig data, in addition to the genetic and pseudo-permanent environmental effects.

ABW and LS were defined as traits of the sow/doe and analyzed using the previously described multiple-trait SAD model with successive time points at each farrowing/kindling. Data were also analyzed using a multiple-trait RR model in order to provide a comparison of the SAD multiple-trait model with the most currently used method. Selection of the degree of the Legendre polynomials for the permanent and genetic effects was performed for each trait by comparing nested single-trait RR models using the log likelihood ratio test. Then, multiple-trait RR models were applied to the data using the selected degree of polynomials for each trait. Goodness-of-fit of the SAD and RR models to the data were compared using the AIC.

## Results

In rabbits, model selection for the antedependence relationship using single-trait analysis showed that the most appropriate SAD models were SAD 111 for the animal genetic effect on both traits (LS and ABW), and SAD 100 and SAD 111 for the pseudo-permanent environmental effect on LS and ABW, respectively. These antedependence characteristics were retained for the multiple-trait model. Using the multiple-trait SAD model, the same cross-antedependence characteristics were selected for the genetic and permanent environmental effects, that is a recursive relationship (i.e. $c > \eta$, in other words, LS random effects are not functions of ABW random effects), $c^{{\prime }} = \eta^{{\prime }} = 0$ (i.e. ABW random effects at parity $j$ are functions of LS random effects at parity $j$) and a degree 2 for the cross-antedependence function of time, which resulted in 20 parameters to model ${\mathbf{U}}$ and ${\mathbf{P}}$. Let $LS_{i} \left( j \right)$ and $ABW_{i} \left( j \right)$ be the LS and ABW of dam $i$ at parity $j$, the selected multiple-trait SAD model then is:

$$LS_{i} \left( j \right) = {\mathbf{x}}_{ij}^{{\prime }} {\varvec{\upbeta}}_{LS} + u_{i} \left( j \right) + p_{LSi} \left( j \right),$$

$$ABW_{i} \left( j \right) = {\mathbf{x}}_{ij}^{{\prime }} {\varvec{\upbeta}}_{ABW} + \nu_{i} \left( j \right) + p_{ABWi} \left( j \right),\;{\text{with}}$$

$$u_{i} \left( j \right) = \theta_{u,1j} u_{i} \left( {j - 1} \right) + e_{u,i} \left( j \right),$$

$$\nu_{i} \left( j \right) = \theta_{\nu ,1j}^{{\prime }} \nu_{i} \left( {j - 1} \right) + \delta_{\nu ,0j}^{{\prime }} u_{i} \left( j \right) + e_{\nu ,i} \left( j \right),\;{\text{and}}$$

$$p_{LS,i} \left( j \right) = \theta_{p,1j} p_{LS,i} \left( {j - 1} \right) + e_{pLS,i} \left( j \right),$$

$$p_{ABW,i} \left( j \right) = \theta_{p,1j}^{{\prime }} p_{ABW,i} \left( {j - 1} \right) + \delta_{p,0j}^{{\prime }} p_{LS,i} \left( j \right) + e_{pABW,i} (j).$$

In pigs, the SAD 101 model was retained for the within-trait antedependency for the genetic and pseudo-permanent environmental effects for LS, and SAD 111 and SAD 101 for the genetic and pseudo-permanent environmental effects for ABW, respectively. Recursive cross-antedependence functions of order 1 with $c^{{\prime }} = 0$, degree 0 and 1 were selected for the genetic and pseudo-permanent environmental effects, respectively. The random contemporary group effects of the two traits were independent.

After model selection, the single trait RR models selected in pigs included a Legendre polynomial of degree 1 for the genetic effects and a constant permanent effect over time for both traits. In rabbits, the best single trait RR model was of degree 1 for both the genetic and permanent environmental effects for both traits. Unfortunately, the multiple-trait extension of these models did not converge in rabbits. Thus, the multiple-trait RR model considered for rabbits consisted, as for pigs, of a polynomial function of degree 1 for genetic effects only.

In pigs, the AIC for the multiple-trait SAD and RR models were 66,319 and 66,326, respectively. For rabbits, the AIC were 70,684 and 70,911 for the multiple-trait SAD and RR models, respectively.

Heritability estimates with the SAD model for the two traits are in Table 2. Heritability estimates were moderate for all traits at all parities, with the exception of LS at parity 1 in rabbits, which had a lower heritability estimate, i.e. 0.09. Heritability estimates for ABW were generally higher than for LS. In pigs, heritability estimates were quite stable across parities, ranging from 0.19 to 0.25 for LS and from 0.29 to 0.35 for ABW. In rabbits, heritability estimates tended to increase with parity for both traits, ranging from 0.09 to 0.29 for LS and from 0.23 to 0.39 for ABW. In pigs, heritability estimates obtained with the RR model tended to be slightly lower than those obtained with the SAD model, ranging from 0.18 to 0.28 for LS and from 0.22 to 0.31 for ABW. In rabbits, compared to the SAD model, heritability estimates obtained with the RR model were slightly lower for LS (ranging from 0.08 to 0.25) and higher for ABW (ranging from 0.24 to 0.42).

**Table 2:** Heritability estimates obtained with the SAD model in pigs and rabbits

Parity	Pigs		Rabbits	
	Litter size	Average BW	Litter size	Average BW
1	0.21	0.29	0.09	0.23
2	0.25	0.33	0.19	0.26
3	0.24	0.35	0.27	0.32
4	0.22	0.33	0.29	0.37
5	0.19	0.30	0.24	0.39

**Fig. 1:**
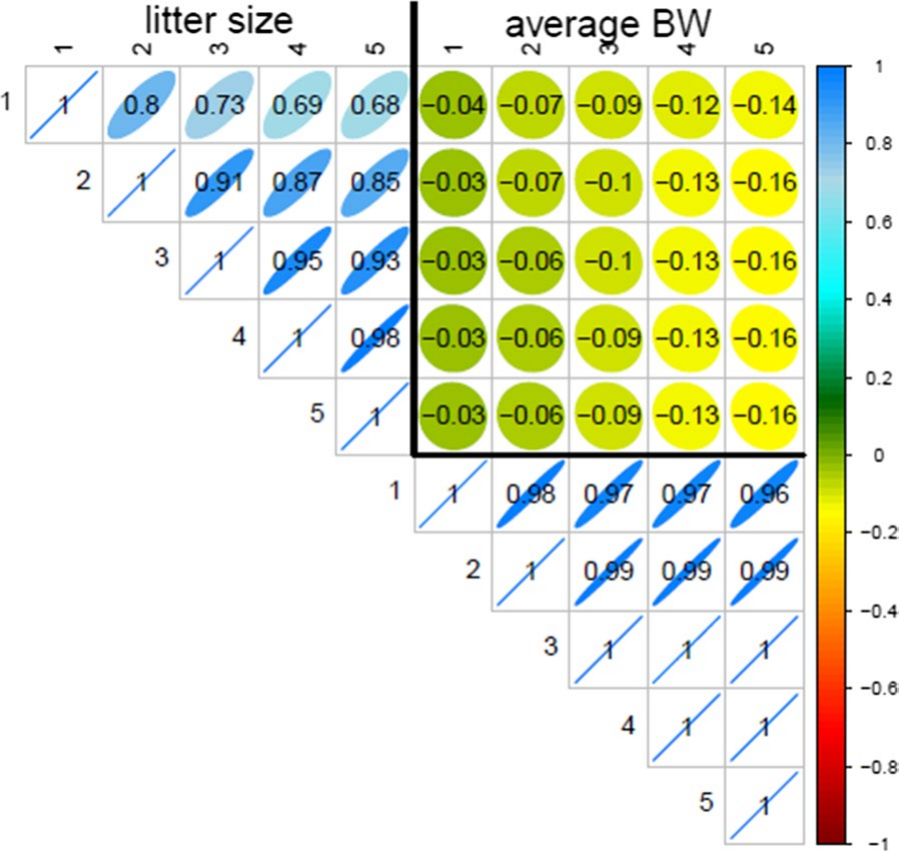
Genetic correlations estimated with the SAD model in pigs

**Fig. 2:**
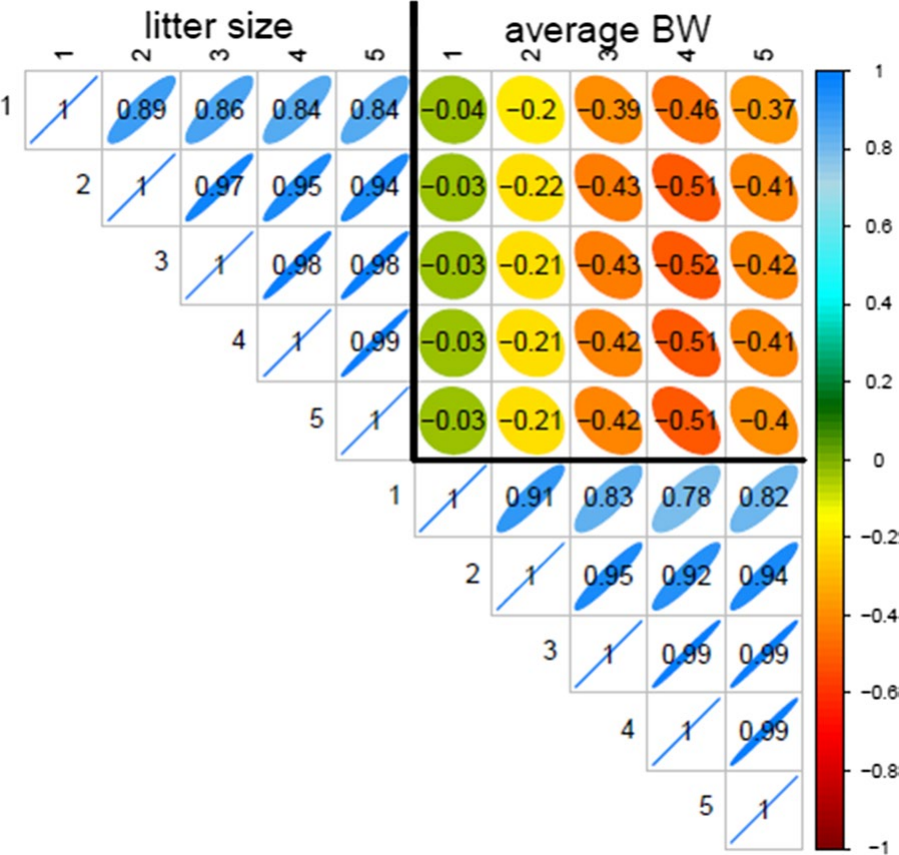
Genetic correlations estimated with the SAD model in rabbits

Genetic correlation matrices estimated with the multiple-trait SAD models are shown in Figs. 1 and 2 for pigs and rabbits, respectively. Estimates of the genetic correlation between LS tended to decrease as the distance between parities increased, the decrease being slightly more pronounced for pigs than for rabbits (0.84 versus 0.68 between parities 1 and 5). The same general pattern, i.e. a decrease of the estimated genetic correlation between more distant parities, was observed for ABW in rabbits. In pigs, estimates of the genetic correlations between ABW at different parities were high, the minimum being 0.96 between parities 1 and 5. Estimates of the genetic correlation between LS and ABW were negative in both species. In general, we observed an increase of the genetic antagonism between the two traits with the parity level for ABW regardless of the parity level for LS.

**Fig. 3:**
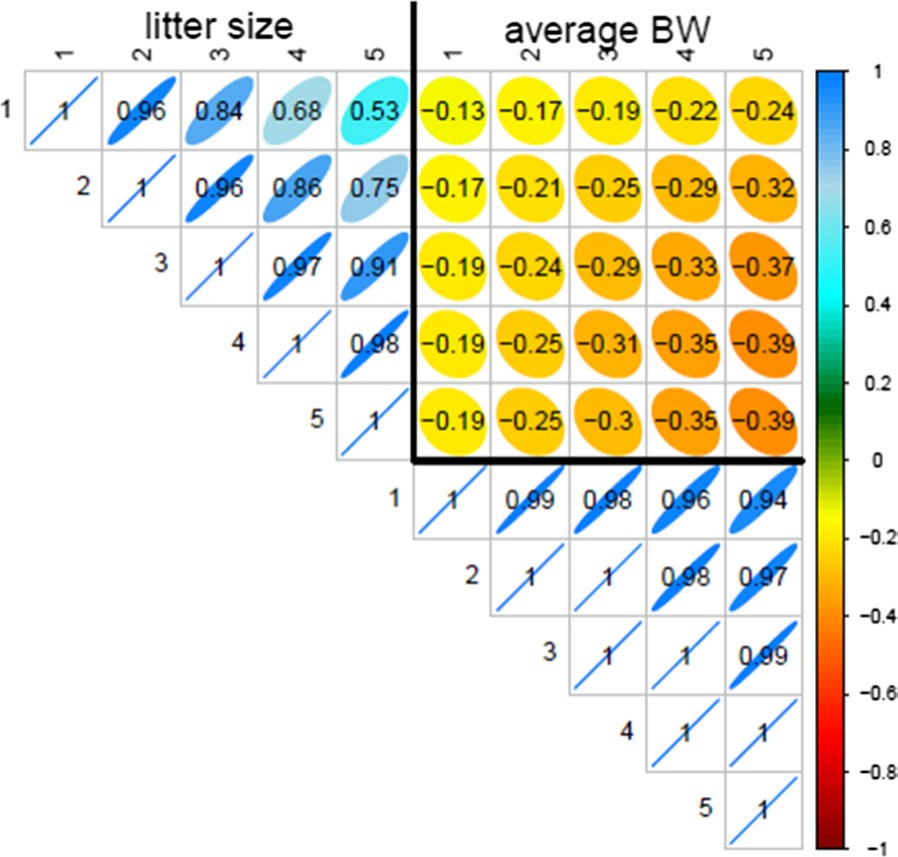
Genetic correlations estimated with the RR model in pigs

The genetic correlation matrix estimated with the RR model for pigs is in Fig. 3. The general pattern of these correlation estimates was similar to the one obtained with the SAD model for both species but with a more pronounced genetic antagonism between the two traits in pigs and less pronounced in rabbits (result not shown, the lowest correlation between traits in rabbits was equal to −0.34).

**Fig. 4:**
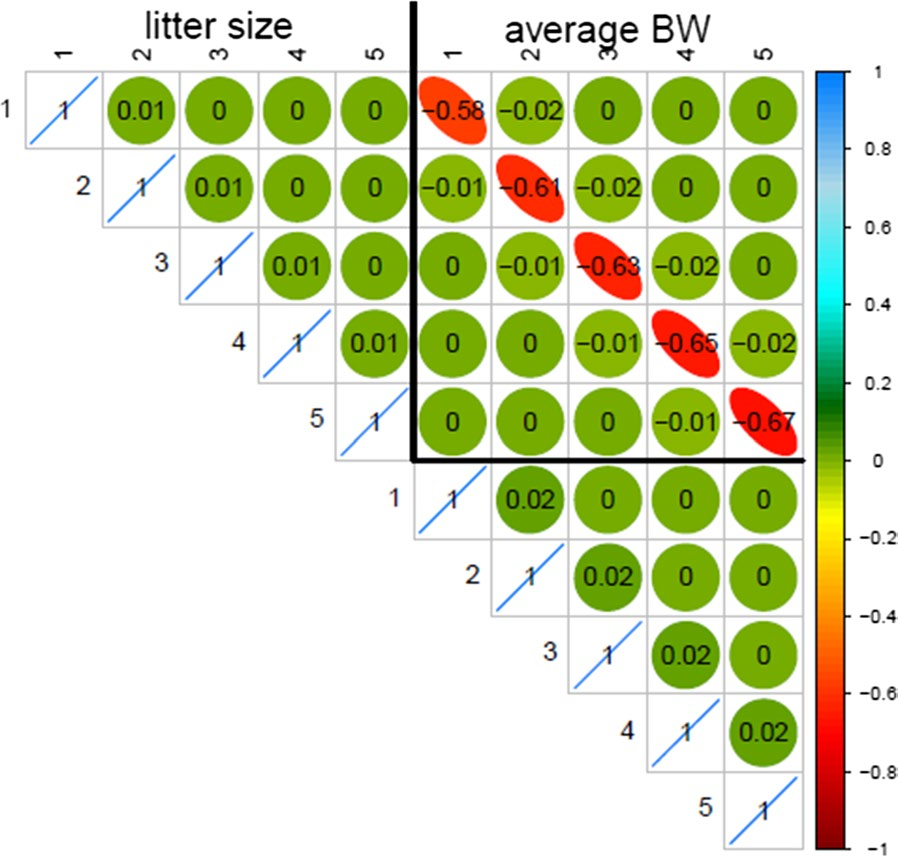
Pseudo-permanent effect correlations estimated with the SAD model in pigs

**Fig. 5:**
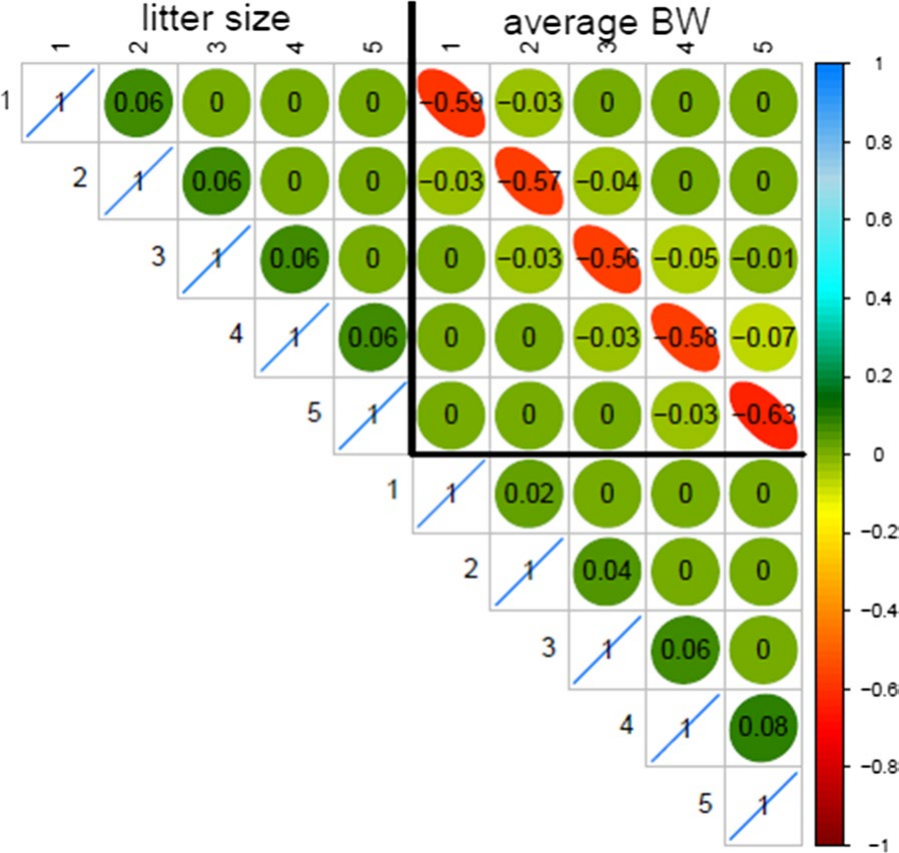
Pseudo-permanent effect correlations estimated with the SAD model in rabbits

**Fig. 6:**
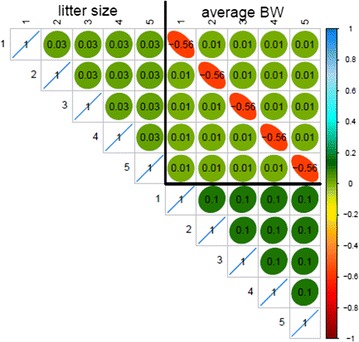
Pseudo-permanent effect correlations estimated with the RR model in pigs

Correlation matrices for the pseudo-permanent environmental effects obtained with the multiple-trait SAD model are in Figs. 4 and 5 for pigs and rabbits, respectively. Estimates of the correlations between pseudo-permanent environmental effects at different parities within-trait were close to 0. The same across-trait pattern of estimates of correlations between pseudo-permanent environmental effects was observed for both species: a high negative correlation between the pseudo-permanent environmental effects of the two traits for the same parity level (ranging from −0.57 to −0.67) and correlations close to 0 otherwise. The same general pattern of correlations was obtained with the RR model for pigs (Fig. 6) and rabbits (result not shown).

## Discussion

We propose in this article a multiple-trait SAD model that takes the within-trait and cross-trait correlations over time into account. A first multiple-trait extension of the SAD model was proposed a few years back by Jaffrézic et al. [9], however our multiple-trait SAD model differs from theirs regarding the assumption made about the innovation covariance matrix. For their extension, Jaffrézic et al. [9] assumed that the error terms ${\mathbf{e}}_{1} \left( t \right)$ and ${\mathbf{e}}_{2} \left( t \right)$ are correlated as a function of time. To insure identifiability of the parameters, our assumption is that these error terms are independent, except at time $t_{1}$, when they can be correlated if $c > 0$ and $c^{{\prime }} > 0$ in Eq. (1).

We suggest a two-step procedure for selecting the order of (cross-)antedependence and degree of the polynomial functions: within-trait selection of the antedependence parameters using a single-trait SAD model, followed by selection of the cross-antedependence parameters using the multiple-trait SAD model. This same two-step approach was performed by Jaffrézic et al. [9]. In the multiple-trait SAD model, selection of the order of cross-antedependence may differ according to the goal of the analysis. If the goal is to estimate the correlation between traits, our experience showed that a recursive cross-antedependence with zero as starting point and order 1 (i.e. $c^{{\prime }} = \eta^{{\prime }} = 0$) is generally sufficient to model all forms of correlation across traits. In such cases, only the degree of the cross-antedependence function needs to be selected by comparing nested models using the likelihood ratio test. Conversely, if the goal of the analysis is to study causal relationships between traits, it seems necessary to define, a priori, the general pathway between traits, as in SEM (resursiveness, simultaneity), to fit the order of the cross-antedependence in the multiple-trait SAD model to match the pathway. Then, the degree of the polynomial function for each cross-antedependence parameter is selected by comparing nested models using the likelihood ratio test. In such situations, antedependence parameter values are of interest in addition to the ${\mathbf{U}}$ and ${\mathbf{P}}$ covariance matrices.

Similar to a RR model, the number of breeding values predicted per animal with a SAD model equals the number of time points. Eigen decomposition (eigenvalues, eigenvectors) of the genetic covariance matrix obtained with the SAD model can help to determine which linear combination of breeding values can be used for genetic selection purposes. Nonetheless, given the way the SAD covariance matrix is structured, a decomposition in eigenfunctions (continuous function of time) similar to that proposed for RR models [26] is certainly not feasible.

We applied the multiple-trait SAD model to study the correlation between LS and ABW over parities in two species. For this purpose, we used a recursive cross-antedependence relationship. To study the causal relationship between these two traits with a SEM, Varona et al. [12] proposed a one-way causal path that established an effect of LS on ABW within parity as the most likely general pathway between LS and ABW. If our goal was to study the causal relationship between traits, similar to Varona et al. [12]. we would have needed to add cross-antedependence parameters to the SAD model that links the random parameters of ABW at time $t$ to those of LS at time $t + 1$. As in previous studies, we considered LS and ABW as traits of the dam [12, 13].

Heritability estimates for LS were higher than the values of about 0.10 that were reported in previous studies [27–31] but were consistent with the total heritability for LS reported for the same pig breed by Kaufmann et al. (0.24) [32] and for rabbits by Nagy et al. (0.11–0.31) [33]. The higher heritabilities obtained in the current study can be explained by the fact that heritabilities estimated with a model that assumes different traits over time are generally higher than those obtained by using a simple repeatability model [34]. An increase of the heritability of LS with parity similar to that observed here was previously reported for both species [28–30], however it was not observed in pigs by Lukovic et al. [35] using records over 10 parities. Our heritability estimates for ABW were consistent with those reported by Hermesch in pigs (0.31) [13] but higher than those reported by Varona et al. in pigs (0.23) [12] and Bolet et al. [36] (0.04) or Garreau et al. [37] (0.06) in rabbits.

On the one hand, the decrease in the within-trait genetic correlation with distance between measurements (parities) is a result frequently reported in the literature [38, 39] and the genetic correlation matrix reported by Hanenberg et al. [28] for LS in Dutch Landrace pigs is close to the estimates obtained in our study. On the other hand, estimates of the genetic correlation for ABW reported for Australian pigs tended to be lower than obtained in the current study, but their standard errors were high [40]. It is generally recommended to consider LS and ABW at first parity as different traits from the performances at later parities [28, 40]. The same conclusion can be drawn from our results, except for ABW in pigs, which can be considered as a repeatable trait, the lowest genetic correlation value being 0.95 between parity 1 and 5.

Regarding cross-correlations, our results showed that the general trend was an unfavorable genetic correlation between LS and ABW (slightly negative in pigs and more strongly negative in rabbits). Several studies have reported the same negative genetic correlation between these traits in pigs [31, 40, 41]. Even if the trend was less clear in pigs, we observed the same cross-correlation tendency over parities: ABW tended to be more negatively correlated to LS in late parities than in first parity. In other words, if one considered that LS occurs before ABW and studies the relationship between successive traits (i.e. ABW at time $t - 1$ has an “effect” on LS at time $t$ which has an “effect” on ABW at time $t$ and so on), our results show that the antagonism between successive traits increases with parity. This could be considered as an increasing adaptation of traits to their environmental conditions with time, LS at time $t$ being part of the environment for ABW at time $t$, which in turn is the environment to which the animal has to adapt its LS at time $t + 1$ and so on. Hermesch et al. [40] also studied cross-correlations over parities in pigs and did not find any trend, but their estimates had large standard errors.

The close to 0 correlations that we found between pseudo-permanent effects of the same trait were previously reported between first and second parities in rabbits [30] and pigs [40]. Consistent with our results, Hermesch et al. [40] reported a strong negative correlation between the pseudo-permanent effects of the two traits at the same parity and no correlation between traits at different parities. In our model, the so-called pseudo-permanent environmental effect combines the dam characteristics that are not under genetic control and persist over parities and the environmental factors that are not taken into account in the model because not measured/observable. The correlation between pseudo-permanent environmental effects at different parities was extremely low. Thus, the pseudo-permanent effects probably mainly reflect specific animal parity-related factors that are not taken into account in the model or environmental factors that affect traits differently during the reproductive period. Given the high negative correlation between the pseudo-permanent environmental effects between traits, these unobservable factors have opposite effects on LS and ABW.

We compared results obtained with the multiple-trait SAD and RR models. The latter is the approach most often used for analyzing longitudinal data in genetic studies. Probably due to the small number of repetitions, the best RR model selected in pigs and the multiple-trait RR model that converged in rabbits only considered a Legendre polynomial of degree 1 for genetic effects. Thus, with the same number of parameters in pigs (16), the RR model was less flexible than the multiple-trait SAD model to model the variance covariance matrix of environmental effects. Comparison of AIC values showed that the multiple-trait SAD model provided a better fit to the data than the RR model in both species. The same conclusion has been drawn in previous studies on other traits [8, 9]. Results obtained with RR models were essentially consistent with those of the multiple-trait SAD model, which enables us to be confident in the estimations obtained with the SAD approach.

## Conclusions

In this paper, we have outlined a multiple-trait SAD model to simultaneously analyze repeated measurements of several traits. This flexible approach was developed to provide an advantageous approach to model the covariance matrix of random terms with few parameters. When used to study relationship between LS and ABW over five parities in two species, the multiple-trait SAD model showed that a total of 16 or 20 parameters was sufficient to model the random term covariance matrices. This is much less than the number of parameters required to obtain estimates when unstructured covariance matrices are assumed for ${\mathbf{U}}$ and ${\mathbf{P}}$ (up to 110 parameters). Furthermore, we showed that multiple-trait SAD models provide a better fit to the data than multiple-trait RR models. We offer a freely-available online Fortran program that can be used to implement this SAD model in ASReml.
